# Disentangling the influence of reservoir abundance and pathogen shedding on zoonotic spillover of the *Leptospira* agent in urban informal settlements

**DOI:** 10.3389/fpubh.2024.1447592

**Published:** 2024-09-18

**Authors:** Nirali Soni, Max T. Eyre, Fábio N. Souza, Peter J. Diggle, Albert I. Ko, Mike Begon, Roger Pickup, James E. Childs, Hussein Khalil, Ticiana S. A. Carvalho-Pereira, Arsinoê C. Pertile, Mayara Carvalho, Daiana de Oliveira, Nivison Nery, Emanuele Giorgi, Federico Costa

**Affiliations:** ^1^Centre for Health Informatics, Computing, and Statistics, Lancaster University Medical School, Lancaster, United Kingdom; ^2^London School of Hygiene and Tropical Medicine, London, United Kingdom; ^3^Institute of Collective Health, Federal University of Bahia, Salvador, Bahia, Brazil; ^4^Instituto Gonçalo Moniz, Fundação Oswaldo Cruz, Ministério da Saúde, Salvador, Bahia, Brazil; ^5^Department of Epidemiology of Microbial Diseases, School of Public Health, Yale University, New Haven, CT, United States; ^6^Department of Evolution, Ecology and Behaviour, University of Liverpool, Liverpool, United Kingdom; ^7^Division of Biomedical and Life Sciences, Faculty of Health and Medicine, Lancaster University, Lancaster, United Kingdom; ^8^Department of Wildlife, Fish and Environmental Studies, Swedish University of Agricultural Sciences, Umeå, Sweden

**Keywords:** leptospirosis, rat, human, pathogen, shedding, abundance

## Abstract

Rats are major reservoirs for pathogenic *Leptospira*, the bacteria causing leptospirosis, particularly in urban informal settlements. However, the impact of variation in rat abundance and pathogen shedding rates on spillover transmission to humans remains unclear. This study aimed to investigate how spatial variation in reservoir abundance and pathogen pressure affect *Leptospira* spillover transmission to humans in a Brazilian urban informal settlement. A longitudinal eco-epidemiological study was conducted from 2013 to 2014 to characterize the spatial distribution of rat abundance and *Leptospira* shedding rates in rats and determine the association with human infection risk in a cohort of 2,206 community residents. Tracking plates and live-trapping were used to measure rat abundance and quantify rat shedding status and load. In parallel, four sequential biannual serosurveys were used to identify human *Leptospira* infections. To evaluate the role of shedding on human risk, we built three statistical models for: (1) the relative abundance of rats, (2) the shedding rate by individual rats, and (3) human *Leptospira* infection, in which “total shedding”, obtained by multiplying the predictions from those two models, was used as a risk factor. We found that *Leptospira* shedding was associated with older and sexually mature rats and varied spatially and temporally—higher at valley bottoms and with seasonal rainfall (December to March). The point estimate for “total shedding” by rat populations was positive, i.e., *Leptospira* infection risk increased with total shedding, but the association was not significant [odds ratio (OR) = 1.1; 95% confidence interval (CI): 0.9, 1.4]. This positive trend was mainly driven by rat abundance, rather than individual rat shedding (OR = 1.8; 95% CI: 0.6, 5.4 vs. OR = 1.0; 95% CI: 0.7, 1.4]. Infection risk was higher in areas with more vegetative land cover (OR = 2.4; 95% CI: 1.2, 4.8), and when floodwater entered the house (OR = 2.4; 95% CI: 1.6, 3.4). Our findings indicate that environmental and hydrological factors play a more significant role in *Leptospira* spillover than rat associated factors. Furthermore, we developed a novel approach combining several models to elucidate complex links between animal reservoir abundance, pathogen shedding and environmental factors on zoonotic spillover in humans that can be extended to other environmentally transmitted diseases.

## 1 Introduction

Leptospirosis is a zoonotic disease that is responsible for 1 million annual cases and 60,000 annual deaths worldwide, making it a global health concern (1). Symptoms vary, and can be mild for most people, but around 10% of individuals experience severe symptoms such as Weil's disease and pulmonary hemorrhage (2). In addition, the disease is estimated to lead to an annual global loss of 2.9 million disability adjusted life-years (3). The bacterial genus *Leptospira* comprises many species that can cause severe disease in humans, of which *L. interrogans*, is the most notable (4).

Norway rats, *Rattus norvegicus*, are one of the main reservoirs for the agents of leptospirosis. Once infected, they remain largely asymptomatic, whilst releasing pathogenic leptospires in their urine for the rest of their lives (5–7). Other animals have also been implicated [e.g., livestock, bats as well as companion animals (8, 9)]. The disease is transmitted to humans through breaks in the skin coming into direct contact with infected animals, or with environments contaminated with the pathogenic leptospires (5, 10).

Although leptospirosis is widespread globally, its incidence is higher in tropical than in temperate climates and the highest disease burden is faced by those in low and middle-income countries (1, 3). Exposure to heavy rainfall and flooding are major risk factors for acquiring the disease, by increasing contact with contaminated water, leading to a sustained increase in transmission to humans (11, 12). Other risk factors include poverty, poor sanitation, overcrowding, inadequate hygiene standards and access to healthcare, putting those living in urban slum environments at a greater risk of contracting the disease, thus adding to the already significant inequalities faced by these communities (13).

It has been hypothesized that higher rates of leptospire shedding by subpopulations of rats into the environment can lead to higher rates of infection in humans (14, 15). Fine geographical scale variation in human infection not explained by the environmental components (such as flooding) in previous studies (16) could be associated with variation in *Leptospira* shedding. Additionally, other disease systems also show that shedding by subpopulations of animals can play an important role in driving human infections in the form of super-spreaders, although this is not known for leptospirosis (17, 18). Previous work found that rat abundance is an important risk factor for the disease, but did not explore the role of variation in shedding rates (19).

The mechanisms by which shedding can lead to spillover infection are not well-elucidated. Specifically, factors driving urban residents' infection include the combination of physical environmental (e.g., elevation, rainfall), behavioral (e.g., occupation, preventative measures), and reservoir population (e.g., *Leptospira* prevalence in rat reservoir, rat abundance) factors that drive reservoir shedding rates (pathogen pressure), as well as the association between pathogenic leptospire concentration in the environment and human infection risk. It has been proposed that zoonotic pathogens require alignment of numerous barriers and factors for spillover infection to happen (20). Therefore, gaining a better understanding of variation in shedding rates by individual rats and within the environment will delineate the dynamics of zoonotic spillover at the human-animal-environment interface and inform targeted One Health interventions to protect the most marginalized populations through reductions in disease risk.

Here, we aimed to investigate the extent to which spatial variation in reservoir abundance and pathogen pressure promote increased *Leptospira* spillover transmission to humans in the setting of an urban informal settlement in Brazil. This was done by building separate statistical models to predict rat abundance and individual rat shedding. These two metrics were multiplied to estimate “total shedding”, our risk factor of interest, which we then used as an explanatory variable for a human infection model. This extends previous work, which used rat abundance as a proxy for the environmental risk of infection (19). Here, we explored a novel approach of combining the results from several statistical models to determine if adding individual shedding to rat abundance can further explain the variation in infection risk and its relative contribution compared to social and environmental factors.

## 2 Materials and methods

### 2.1 Study site

This study was carried out in Pau da Lima, Salvador, Brazil. Pau da Lima is an urban informal settlement comprised of informal housing situated in four valleys, and in an area of 0.46 km^2^ (16) with a high leptospirosis incidence, estimated to be 51.4 per 1,000 annual follow-up events (19). On-going eco-epidemiological research has taken place in this area for the last two decades, involving serial annual to biannual surveys of the community, as well as ecological studies (16, 21, 22). The site has been described previously (21). The majority of residents (85%) do not have legal titles for their homes. The area also has poor infrastructure and sanitation, such as steep inclines and open sewers (16, 22). A 2003 study census found the population to be just over 14,000 residents, from 3,600 households (16, 21, 22).

### 2.2 Data collection

#### 2.2.1 Rodent trapping and leptospiral shedding data

Data on spatial variation in rat abundance was collected using an ecological cross-sectional study, carried out from October to December 2014 (23). Track plates were placed at 385 spatially randomized individual locations, at each of which data for environmental variables were available (19). Collection took place during two consecutive 24-h periods. This technique has previously been found to be highly correlated to signs of rodent infestation (24). Five plates were placed at each location, in the configuration of the “5” face on a dice (24). However, at some locations, fewer than five plates were useable due to theft or damage. Details on data collection have been described previously (19, 22).

In addition, to collect data on individual rat characteristics, trapping data was collected between 2013 and 2014 during four consecutive campaigns—two in the dry season and two in the wet season (campaign 1 May-August 2013; campaign 2 October-December 2013; campaign 3 March-July 2014; campaign 4 September-December 2014). The study site was sampled systematically, using Tomahawk live traps for four or six consecutive nights, and a total of 490 rats (*Rattus novergicus*) were captured. Caught rats were geolocated, individual and surrounding environmental characteristics were recorded, and urine was collected. The population characteristics of this population and details of capture and extraction of urine have been described previously (25). *Leptospira* concentration in urine was tested using qPCR targeting the gene LipL32, which is present in pathogenic leptospires (15, 25). The assumptions made here are detailed in the discussion, but briefly, shedding estimates were assigned to the point and time of capture, and shedding rates were assumed to be constant and consistent.

#### 2.2.2 Human *Leptospira* infection

Alongside the rat data collection, human infection data was collected during a prospective ongoing cohort study carried out in Pau da Lima. Here, we used data from the 2013–2014 surveys (collection 1 January-April 2013; collection 2 August-December 2013; collection 3 January-June 2014; collection 4 August-October 2014). All participants meeting the inclusion criteria were invited to join. Those included being at least 5 years of age and sleeping in the study household for at least 3 nights in the previous week. Data was collected at 6-month intervals, comprising an interviewer-administered questionnaire and a serosurvey, where blood samples were taken (16, 26). This dataset included multiple socio-demographic and environmental factors, and potential sources of contamination, including sex, age, employment, details of the home, and exposures such as rainfall experienced.

Blood samples collected during household visits were processed to determine if infection occurred in the 6-month period preceding a measurement, using a microscopic agglutination test (MAT) to observe agglutination of *Leptospira* specific antibodies (16, 21). The reference were *Leptospira interrogans* serogroup Icterohaemorrhagiae (serovar Copenhageni) and *Leptospira kirschneri* serogroup Cynopteri and serial dilutions were performed to determine the largest dilution titer (16, 21). The outcome was defined as either a seroconversion (i.e., antibody agglutination change from seronegative to over 1:50) or at least a 4-fold increase in serovar-specific antibodies between the two paired samples. The result was classed as a seroconversion if this threshold was met for either or both serogroups (27). Moreover, samples displaying negligible reactivity, indicated by an antibody titer below 1:50 for the tested serogroups, were classified as negative. The outcome also accounted for titer decay between two paired measurements using the methodology described by Owers Bonner et al., to avoid the misclassification of individuals because of titer decay occurring between measurements at long time-intervals (28).

### 2.3 Ethics

The protocols used for the ecology studies were approved by the Oswaldo Cruz Foundation, Salvador, Brazil (protocol number 003/2012). Written informed consent was gained from enrolled participants, and procedures were approved by the Institutional Review Boards of the Oswaldo Cruz Foundation and Brazilian National Commission for Ethics in Research, Brazilian Ministry of Health (CAAE: 01877912.8.0000.0040) and Yale University School of Public Health (HIC 1006006956).

### 2.4 Statistical analysis

**Figure 1:**
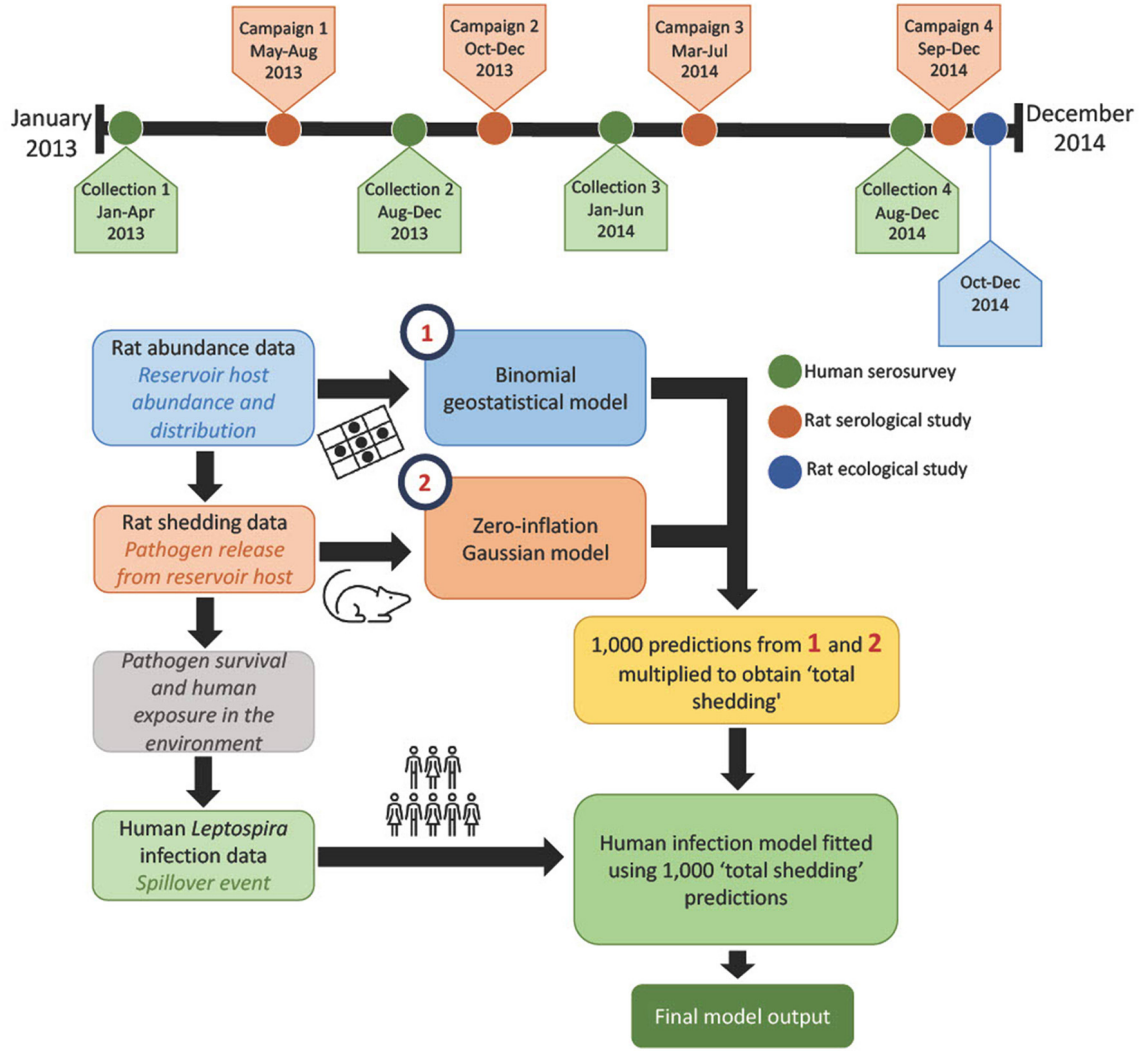
Shows the process followed here, using each layer of data, and a timeline of when data collection took place. Maps showing the results from the models can be found in Figure 2.

Here, we define the modeling framework to estimate the association between the pathogenic leptospires in the environment and human infections. Briefly, total leptospire shedding (defined hereafter as “total shedding”) was estimated by multiplying predictions of rat abundance and individual rat shedding, as shown in Figure 1.

#### 2.4.1 Rat abundance model

A binomial geostatistical model was built to estimate the abundance of rats in the study area, and to account for residual spatial correlation (see Supplementary material 1 for details) in the number of track plates at each measured location showing evidence of rat markings (from 0 to 5) out of the total number of plates at each point (24, 29). To select the covariates for the rat abundance model, all relevant variables were included in a multivariable logistic regression model, and backward-elimination was performed to choose the most suitable model. The Akaike information criteria (AIC) value was used to rank the models, and the one with the lowest value was chosen (30). Where 2 models were within 2 AIC of each other, the simpler model was chosen. Parameter estimation was carried out using maximum likelihood estimation via a Markov Chain Monte Carlo approximation of the likelihood function of the geostatistical model (more details are found in Supplementary material 1) (29).

#### 2.4.2 Rat shedding model

All rats (PCR-positive and -negative for *Leptospira*) were included in the analysis. The outcome was the log-base-10-transformed leptospire load shed by each rat measured as genome equivalents (GEq) of leptospiral DNA per ml of urine. A zero-inflated Gaussian model was used to model the outcome, to account for the excess number of zeroes due to the sampling of seronegative rats that do not shed leptospires. This model consists of two components: one that models the probability of a rat being seropositive as a logistic regression; a second that models the amount of shedding by seropositive rats using a linear mixed model.

For the modeling of each of these two components, exploratory analysis was carried out to determine the relationship between different explanatory variables and the outcome for the Gaussian portion and modeled using linear regression. Only the seropositive rats were used to carry out this exploratory analysis. The covariates were divided into two broad categories: individual rat characteristics and environmental factors. Univariable analysis was carried out to assess the relationships between the outcome and each variable. Because of the large number of covariates available, all variables with an association of *p*-value < 0.05 were considered, as well as those with established links from the literature. Plots were used to visually determine the relationship of the variables with the outcome, and how they should be included into the model. Again, all potential variables meeting the aforementioned criteria were included in a multivariable linear regression model, and backward-elimination was performed. As before, the AIC value was used to select the best model (30). Details can be found in Supplementary material 2 and Supplementary Figure 1.

Of the covariates analyzed, sex, evidence of sexual activity and wounds were categorical variables. The ratio of length and weight was used as a proxy for rat age to categorize rats as juvenile, young adult or adults (15). The environmental variables included were elevation, proportion of impervious landcover, valley, and distance to sewers; all but valley were continuous measurements. Temporal trends were accounted for by including a log-linear time trend, with a change in the slope after the twelfth month (December 2013; Supplementary Figure 1J). This choice was informed through exploratory analysis of the data. An empirical variogram was plotted and showed no evidence for residual spatial variation (see Supplementary Figure 2 for details); therefore, a geostatistical model was not used. An unstructured random-effects term at cluster-level was included into the fixed part of the zero-inflation Gaussian model. These terms were assigned to each rat according to their location of capture.

The zero-inflated part was modeled as a binomial outcome, where the outcome was the serostatus of the rat (seropositive or seronegative depending on the PCR result). The same process as before was followed to decide which variables should be included in this portion of the model. To choose the model, we favored parsimony as well as low AIC and due to collinearity between variables, we chose the simplest model which included rat age only (31). Details can be found in Supplementary material 2.

#### 2.4.3 Abundance and shedding predictions

Predictions of rat abundance were made over a 2.5 by 2.5 meters regular grid covering the study area, where each pixel was assigned a value for elevation (29). This process provided 1,000 estimates at each pixel which were summarized by taking the mean and mapped. Predictions of leptospire shedding by individual rats were estimated over the same grid used for the predictions of abundance, for December 2013. To carry out the spatial predictions, we devised the following bootstrap procedure for individual rat characteristics, namely sex, age, and sexual activity which were not available for each pixel and were not found to be spatially correlated. We first generated a random value for each variable at each of the pixels, using the empirical probabilities from the data. Specifically, these were: 0.06 for juvenile, 0.21 for young adult and 0.73 for adult rats; 0.41 for male and 0.59 for female rats; and 0.07 for sexually inactive and 0.93 for sexually active rats. After simulating a value for each of these three rat variables over the regular grid, we generated a predictive map of leptospire shedding. We then repeated this procedure 1,000 times and summarized the predicted leptospire shedding by taking the mean of the 1,000 predictions for mapping.

Finally, the 1,000 predictions from the abundance and individual shedding models were multiplied together to obtain a prediction distribution for the “total shedding” risk factor, which was used as a covariate in the human infection model as explained in Section 2.4.4. As with abundance and shedding, a mean of the repeats was used to map “total shedding”.

#### 2.4.4 Human infection model

The outcome was defined as the occurrence of at least one seroconversion episode (defined earlier) between consecutive timepoints during the study period (in the case of more than one recorded seroconversion by an individual, the most recent result was used). Individuals with missing data were excluded, which resulted in the loss of 20 seroconversions. A logistic regression model was fitted to the human leptospiral infection data. A variogram of the residuals was plotted to determine if there was a need for a geostatistical model, but evidence for this was not found (more details found in Supplementary material 3 and Supplementary Figure 5).

As before, variables were divided into domains that were likely to influence the outcome, based on prior knowledge from the literature: individual socio-demographic factors, home environment, work-related factors, environmental factors, and exposure. Exploratory analysis was carried out to decide which variables should be included in the model, and how they should be included, by carrying out univariate analyses and plotting the covariate against the empirical logit of the outcome variable (Supplementary Figures 3, 4). Within each of these domains, variables with *p*-value < 0.05 were retained in the model.

A logistic regression model was fitted. As before, all variables that had *p*-value < 0.05 in univariable analysis were included, and the final model was chosen following backward-elimination, prioritizing the model with the lowest AIC value, and simplicity (details can be found in Supplementary material 3). A mean of the “total shedding” estimate was used to carry out exploratory analysis.

Individual characteristics included age, sex, literacy and working near a sewer, of which only age was a continuous variable. Of the environmental factors, shedding, elevation, rainfall experienced between paired samples, and landcover were continuous variables, proximity to an open sewer and exposure to floodwater were categorical variables. Landcover was defined as the proportion of vegetative landcover in the surrounding 10 m radius.

To account for the uncertainty in the predictions of “total shedding”, we generated 1,000 predictions and then input these into the human infection model using multiple imputation to fit the model 1,000 times and obtain 1,000 estimates of each regression coefficient. The final output of the model was estimated as the mean of these repeats and the standard error of the regression coefficients was computed using the law of total variance.

We also determined the effect of rat abundance and individual shedding compared to using “total shedding” on human infection by including these separately into the human infection model. This was also done by fitting the 1,000 predictions of abundance and shedding to the outcome in succession, and a mean of the coefficient distributions was taken as the final model output.

All analysis was carried out in R version 4.3.1, using the PrevMap package (29) to fit the geostatistical models.

## 3 Results

### 3.1 Rat abundance model

**Figure 2:**
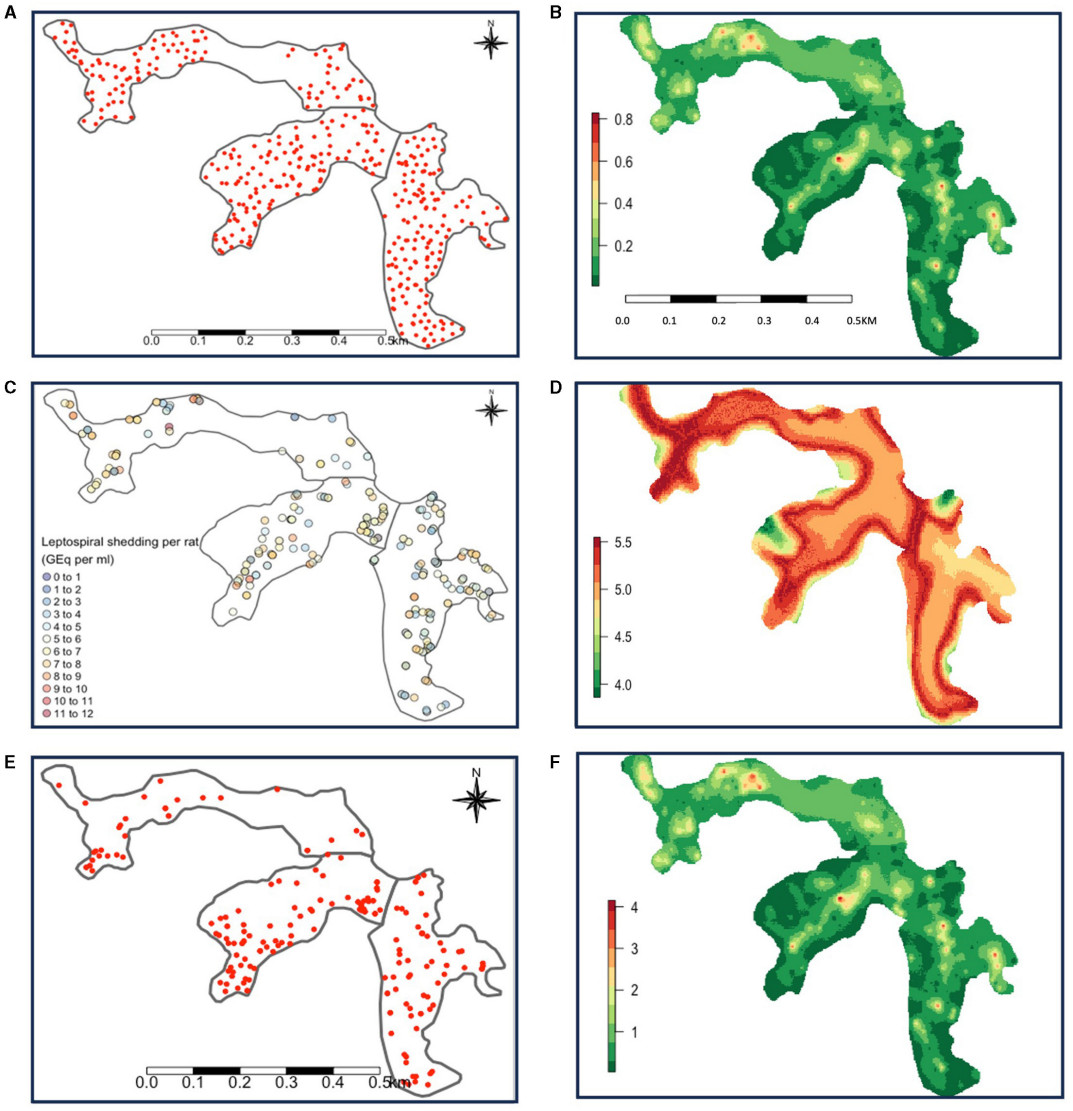
**(A)** Map showing the location of track plates and **(B)** mean rat abundance predictions. **(C)** Map showing the spatial distribution of captured rats and the log leptospire shedding (GEq per ml) by them and **(D)** mean shedding predictions (GEq per ml) by individual rats. **(E)** Map showing the geographical distribution of *Leptospira* seroconversions and **(F)** mean total shedding estimates which show that rat abundance is dominant in driving this variation. Valley 1 is on the far left, valley 2 in the center, and valley 3 on the far right.

Data on rat distribution from track plates was available for 703 individual datapoints, from 385 unique locations across the three valleys. Elevation and valley were both associated with rat abundance. Increasing elevation saw a decreasing trend in rat abundance (Supplementary Table 5). Valleys 2 and 3 had lower abundance than valley 1. Details on the results from the abundance model can be found in Supplementary Table 5. Figure 2A shows the location of the track plates, and Figure 2B shows the mean of the prediction distributions of rat abundance from this model.

### 3.2 Rat shedding model

Data of *Leptospira* infection was available from a total of 489 rats. Of these, 400 were PCR-positive, and 89 were PCR-negative for leptospirosis. A total of 461 (374 seropositive and 87 seronegative) rats were included in the analysis for the shedding model (28 rats were excluded due to missing data). The mean log_10_ leptospire shed in urine by PCR-positive rats was 5.83 [95% confidence interval (CI): 5.66, 6.00] GEq per ml of urine and ranged from 1.8 to 11.8. Figure 2C shows the location of capture of each rat, and the individual leptospire load shed.

Supplementary Table 6 shows stratified rat populations, and the univariable analysis of explanatory factors against the outcome among the PCR-positive rat population included in the analysis. Of the individual rat characteristics, older rats shed higher levels of leptospires, as well as those with evidence of sexual activity (Supplementary Table 6). In addition, shedding was found to be lower in valley 3, and it was also associated with time and distance to sewers (Supplementary Table 6).

A summary of the final shedding model can be found in Table 1. Interestingly, we saw temporal trends in shedding by rats. Log leptospire load decreased by 0.16 per month from May to December 2013, after which it increased by 0.20, holding all other variables constant (Table 1). The opposite trend was observed for elevation, where leptospire load increased initially until elevation 40 m, but decreased thereafter (Table 1). Log leptospire loads were also higher among both young adults and adult rats compared to the reference group of juvenile rats, and when there was evidence of sexual activity (Table 1). Older rats were also more likely to be PCR-positive for leptospirosis (Table 1). Figure 2D shows the mean individual rat shedding predictions.

**Table 1:** Multivariable zero-inflated Gaussian analysis of predictors for individual rat shedding.

**Log shedding**	Beta^*^	95% CI^*^
Intercept	**3.436**	**1.877, 4.995**
**Time**		
January-December 2013 (Month 1–12)	**−0.159**	**−0.231**, **−0.088**
After December 2013 (Month > 12)	**0.196**	**0.087, 0.305**
**Elevation**		
0–40 m	**0.046**	**0.009, 0.084**
>40 m	**−0.108**	**−0.172**, **−0.045**
**Age**		
Juvenile	Ref	–
Young adult	**1.639**	**0.824, 2.453**
Adult	**1.459**	**0.653, 2.265**
**Sex**		
Female	Ref	–
Male	−0.164	−0.469, 0.142
**Evidence of sexual activity**	**0.984**	**0.225, 1.744**
**Probability of sero-positivity**	**OR** ^*^	**95% CI** ^*^
Intercept	**0.801**	**0.462, 1.374**
**Age**		
Juvenile	Ref	–
Young adult	**4.503**	**2.227, 9.318**
Adult	**11.199**	**5.839, 21.901**
**Random-effects**	**Estimate**	**95% CI** ^*^
Variance	**2.091**	**1.849, 2.390**

^*^Bold when *p*-value < 0.05; CI, confidence interval; Ref, reference level; OR, odds ratio.

### 3.3 Human infection model

Data for human seroconversion was available for 2,206 individuals between 2013 and 2014, since infection events could only be defined for individuals with at least one follow-up. This resulted in 4,704 infection event datapoints being collected, of which 261 were infection events (11 positive for Cynopteri, 249 for Copenhageni, 1 for both). After including one result per individual (see Section 2.4.4), and removing individuals with missing data, a total of 2,101 individuals, including 196 *Leptospira* seroconversions remained. Figure 2E shows the distribution of seropositive cases. Figure 2F shows the mean abundance predictions (Figure 2B) multiplied by the mean individual shedding predictions (Figure 2D) to map “total shedding” in Pau da Lima.

The descriptive summaries of the human population, stratified by seroconversion status are presented in Table 2. Of the socio-demographic characteristics, being male, older, and working near a sewer had a higher percentage of infections, whereas a higher social status (being literate) had a lower percentage (Table 2). From the environmental characteristics, living around an increased proportion of vegetative landcover was found to have more infections, as well as living near an open sewer, and having exposure to floodwater (Table 2). Higher levels of leptospire shedding concentrations around the home also had an increased percentage of infections (Table 2).

**Table 2:** Descriptive summaries of individuals included in the human infections cohort, stratified by infection status.

**Characteristic**	**Infection during follow-up events (*N* = 196)**	**No infection during follow-up event (*N* = 1,905)**
	***N*** **or median (% or IQR)**	
**Individual socio-demographic characteristics**		
Age	34 (24)	26 (25)
**Sex**		
Female	85 (43%)	1,094 (57%)
Male	111 (57%)	811 (43%)
**Literacy**		
Illiterate	49 (25%)	249 (13%)
Literate/ < 10 years old	147 (75%)	1,656 (87%)
**Works near sewer**		
No	182 (93%)	1,845 (97%)
Yes	14 (7%)	60 (3%)
**Home and surrounding environmental factors**		
Shedding	0.51 (0.81)	0.44 (0.55)
Elevation (m)	11.11 (10.57)	11.70 (10.56)
Rainfall experienced between paired samples (mm)	887.30 (342.28)	930.50 (299.30)
Landcover	0.13 (0.30)	0.10 (0.24)
**Open sewer**		
No	43 (22%)	585 (31%)
Yes	153 (78%)	1,320 (69%)
**Floodwater entered house**		
No	144 (73%)	1,660 (87%)
Yes	52 (27%)	245 (13%)

*N*, number; IQR, interquartile range.

A summary of the human infection model is shown in Table 3. The point estimate for “total shedding” by rat populations was found to be positive—i.e., risk increased with total shedding, but high uncertainty in estimates meant it was not a significant risk factor for human infections at the conventional 5% level [odds ratio (OR): 1.1, 95% CI: 0.9, 1.4]. The risk of infection initially increased with age (OR: 1.0, 95% CI: 1.0, 1.1), but after the age of 32.5, age was shown to have a slightly protective effect (OR: 1.0, 95% CI: 0.9, 1.0). In addition, male sex had an increased risk of infection (OR: 1.9, 95% CI: 1.4, 2.6), and being literate was protective (OR: 0.7, 95% CI: 0.4, 1.0). Among factors pertaining to surroundings and the environment, infection risk was higher in areas with higher vegetative land cover (OR: 2.4, 95% CI: 1.2, 4.8) and with exposure to floodwater (OR: 2.4, 95% CI: 1.6, 3.4).

**Table 3:** Multivariable logistic regression analysis of predictors for human infection.

**Characteristic**	OR^*^	95% CI^*^
**Intercept**	**0.057**	**0.022, 0.151**
Total shedding	1.110	0.896, 1.374
**Age**		
0–32.5 years old	**1.049**	**1.026, 1.072**
>32.5 years old	**0.958**	**0.927, 0.991**
**Sex**		
Female	Ref	–
Male	**1.946**	**1.433, 2.644**
**Literacy**		
Illiterate	Ref	–
Literate/under 10	**0.656**	**0.445, 0.967**
**Rainfall experienced between paired samples (mm)**	**0.999**	**0.998, 1.000**
**Landcover**	**2.366**	**1.162, 4.815**
**Floodwater entered house**	**2.360**	**1.641, 3.395**

^*^Bold when *p*-value < 0.05; OR, odds ratio; CI, confidence interval; Ref, reference level.

The model including abundance and individual shedding separately rather than using “total shedding” found that rat abundance, rather than shedding by individual rats, was the main driver of the positive association with infection risk (OR: 1.8, 95% CI: 0.6, 5.4), although not significant. Shedding by individual rats was not associated with infection (OR: 1.0, 95% CI: 0.7, 1.4; results shown in Supplementary Table 7).

## 4 Discussion

Here, we used a novel methodology to assess the effect of variations in leptospire concentrations shed by rat populations into the environment on human leptospirosis incidence, to determine their relative importance on the human infection cascade alongside individual and environmental factors. We combined several statistical models to estimate “total shedding” and used this as a risk factor in a human infection model. We were able to model and describe both spatial and temporal shedding rates by individual rats, and better understand the dynamics of “total shedding”, which showed a positive association with disease risk, albeit with considerable uncertainty, that was entirely driven by rat abundance. We also found that human and environmental factors were stronger determinants of risk than local rat shedding, emphasizing the importance of the environmental reservoir. We combined ecological and human epidemiological data to address a key question in leptospirosis research—the contribution of reservoir abundance and pathogen pressure by individual rats in the cascade of spillover *Leptospira* transmission to humans.

The lack of a significant association between total shedding and human infection could suggest that *Leptospira* shedding is a consequence of higher transmission or higher dose inoculum in the rat population at specific moments and places. There may be other factors (e.g., rat movement, *Leptospira* survival in the environment; discussed in limitations) at play that we did not consider, and although we cannot rule out the effect of other animal reservoirs entirely, Norway rats have been found to be the main reservoirs in the study site (32). The high levels of uncertainty in total shedding predictions could be due to high variation in the abundance and shedding data, which could indicate a need for more sampling. Moreover, together our findings of higher shedding rates during rainfall periods and low areas of the valley are congruent with the risk of environmental contamination (33) and higher risk of human infection (11, 16, 21). Rat shedding therefore seems to act as a sentinel of environmental contamination where analysis for environmental contamination (water, soil) is expensive and lab intensive, and has potential to be used as an alternative method.

Rainfall was an important driver of temporal variation in rat shedding rates. Although this association has been studied previously, this was the first study to investigate seasonal trends in the levels of leptospire shedding in Norway rats (15). Several studies have shown a positive association between rainfall and severe leptospirosis case incidence (11, 21, 22). We were interested in determining to what extent this association, at least in part, may have been a result of increased rat shedding. We found seasonal trends, where individual rat shedding decreased from May to December, and then increased, which was in line with the rainy season from December to March. The highest shedding occurred between May and June, which could be a result of a lag between rainfall and an increase in shedding. Although these results do indicate a seasonal trend, it is worth noting that data was only available for one seasonal cycle here, and therefore it is difficult to extrapolate long-term trends. Additionally, this time period also saw unusually low rainfall, which could have affected our results (22).

We also found that elevation could have a compounding effect on disease risk, combining increased shedding, more rats, and environmental exposure. Elevation had a non-linear relationship with rat shedding, which initially increased until an elevation of 40 m and then decreased. We are aware of no research on the link between shedding by individual rats and elevation, but previous studies in the area have found evidence relating elevation to infection risk. A 2016 community-based prospective cohort study investigating determinants of leptospirosis infection found that households at lower altitudes had an increased infection risk (16). This was attributed to lower elevations having a higher risk of environmental exposures, such as mud, open sewers, and flooding, harboring pathogenic leptospires, as well as higher elevations having better drainage systems meaning decreased levels of water accumulation (16). It was also suggested that lower elevations could be associated with lower socio-economic status, although this was most likely a correlation and confounder than cause (16). In addition, rat abundance has also been found to be higher at lower altitudes, whilst also being associated with infection risk, thus augmenting the effect of elevation further (19, 23).

Additionally, older rats shed higher levels of leptospires which could be in line with the assumption that adult rats are more likely to be positive for leptospiral infection. This could explain the link and further strengthen the association between elevation and disease risk as a result of intraspecific competition causing younger rats to be expelled from colonies as they start competing with older rats, forcing them to settle in higher, less favorable areas of the valley (34). This could result in higher elevation areas having additional younger rats—that we found to shed less—driving down the risk.

Furthermore, total shedding risk was more influenced by abundance than by individual shedding (Figure 2). Previous studies have modeled estimates of rat population distributions, and linked them to human infections, where the abundance of rats—defined as “rattiness”—was used as a proxy to quantify the environmental risk of leptospire concentration (19, 23, 32). However, they did not incorporate individual shedding in their models. Here, we used a simpler model to estimate rat abundance, and only included data from track plates, which were found to be the most informative index (23). Doing this, we likely compromised on model fit, but we prioritized simplicity and interpretability, since our outcome of interest was total shedding. Our results highlight that abundance alone may be informative enough to estimate risk and could validate the use of abundance as a proxy for disease risk in future studies, in the absence of shedding estimates as these are not readily available and difficult to obtain. Though neither abundance nor shedding can perfectly capture environmental risk.

In the human infection model, the socio-demographic factors age and sex were associated with human *Leptospira* incidence. These results corroborate existing research, where male sex, and a working age are risk factors for infection, often resulting from increased exposure to contaminated environments, for example at work (13, 16, 21, 22). We used literacy as a proxy for social status and found a protective association between being literate and incidence. Poverty is an established risk factor for leptospirosis (13), and it is worth noting that although this was an informal settlement, there was still variability in levels of literacy, income and employment in the dataset. Other variables that could have been used as proxies for socio-economic status include household income, employment status and employment type. Although some of these variables may have better modeled this relationship, we chose to use literacy because it contained little missing data. Also, education level may be a more holistic representation of an individual's social status compared to income alone.

Of the environmental factors, increased vegetative landcover and exposure to flooding were significant risk factors for disease. This is in accordance with previous research showing increased proximity and exposure to mud and soil to be risk factors for disease, as they can harbor pathogenic leptospires (22). However, those who did and did not experience an infection event had similar rainfall exposures (defined as mm of rain experienced between paired samples). Given the link between increased leptospirosis hospitalisations with flooding events and heavy rainfall, this result seems counterintuitive (11, 21, 35). However, this trend was observed in another study from the same period, which also found that although hospitalisations, and thus serious illness, increased with cumulative rainfall, the risk of sub-clinical disease in the population decreased (22). It was suggested that although heavy rainfall could increase exposure to higher levels of pathogens, leading to higher incidence of severe disease, it could also disturb and mobilize soil and mud around households, thus washing away pathogens and decreasing exposure (22). Therefore, the relationship between the force of infection and serious vs. sub-clinical infection needs further investigation (22). Given this, our variable for flooding exposure (floodwater entering the house) may be a better indicator of estimating exposure alone.

The chance of human spillover transmission by reservoir species occurring is dependent on the alignment of, and overcoming multiple barriers, from host abundance, to pathogen survival, human exposure and immune responses, as suggested by the model presented by Plowright et al. (20). In the context of this model, the uncertainty of our results highlights the complexity of the pathway leading to a spillover event and lend support to the idea that distant sources of contamination such as floodwater and mud may be more important sources of infection, whereas more direct infection from rat urine shed locally may be of less importance.

Previous research on other disease systems has shown that increased shedding by individual reservoir species can result in an increase in human infection risk (18). In the case of *E.coli* O157, this was seen from the existence of super-spreader cattle, which describes individuals of reservoir species that release large numbers of pathogens (18). Consequently, this shows that individuals can have a large impact on disease transmission (17, 18, 36). Conversely, our results indicate that leptospirosis transmission by rats has a different system, which is driven by the population rather than at an individual level. That is to say, the disease risk is not necessarily related to where rats shed but rather human behavior and where you live that is driving risk.

One of the limitations of this study was that the modeled association between total shedding and *Leptospira* incidence was reliant on the fit, quality and assumptions of the individual models for abundance and shedding. Another limitation was that we assigned individuals with a value for “total shedding” based on their household location. This is often used in research; however, people can spend substantial amounts of time in other places, most notably at their place of work, which would also contribute to their total exposure. Against this, a study using GPS tracking to study human mobility in Pau da Lima found that almost 90% of individuals remained within 50 m of their home, which adds face validity to our estimates (37). Similarly, rat shedding estimates were assigned to the point and time of capture of the rats, which is likely not where leptospires accumulate. Moreover, shedding was assumed to be constant and consistent, which again, is likely not entirely accurate. However, given that accurate spatial and temporal estimates of varying shedding by individual rats was not available, these assumptions were necessary. The same applies to *Leptospira* survival in the environment, which was not included in the model, and could have masked temporal trends. However, once again, accurate spatial estimates to do this were not available. In addition, here, we did not account for the dynamicity of rat abundance by using data collected from one survey. As such, our predictions of “total shedding” were estimated at one timepoint, whereas the models were parameterised using data spread over a period of almost 2 years.

These limitations notwithstanding, we have modeled the relationship of leptospire shedding and analyzed its association with human *Leptospira* incidence for the first time in a statistically robust manner. By using a multiple imputation technique, we accounted for some of this uncertainty in our estimates. We have also shown an indication of temporal trends in leptospire shedding by rats. Given these results, spillover transmission is likely multifaceted, and effective prevention strategies will require control of the reservoir population in addition to addressing the structural features of slum settlements that promote transmission.

## 5 Conclusion

We explored a novel approach of combining several statistical models to elucidate the relative contribution of reservoir abundance and pathogenic pressure on human *Leptospira* incidence, and the interplay with other risk factors for the first time and established the importance of the environmental reservoir over shedding by individuals. As well as this, we were able to better understand the temporal trends associated with shedding by individual rats which is important because pathogenic leptospires can be a source of spillover infections to humans.

## Data Availability

Publicly available datasets were analyzed in this study. This data can be found here: Zenodo repository (doi: 10.5281/zenodo.11146445).
