# Supplementary material for: Disentangling the influence of reservoir abundance and pathogen shedding on zoonotic spillover of the Leptospira agent in urban informal settlements
Source: Front Public Health. 2024 Sep 18;12:1447592. doi: 10.3389/fpubh.2024.1447592 (PMC11445007; doi:10.3389/fpubh.2024.1447592)
Supplement: Supplementary file 1 [file Data_Sheet_1.docx]

Supplementary Material

# Details of abundance model

A Binomial model with a logit transformation was used to carry out exploratory analysis, with the outcome as the number of plates with markings on them out of the total. All relevant variables were included, and backwards elimination was used to rank models and choose the final one, prioritising the one with the lowest Akaike information criteria (AIC) (Supplementary Table 1). In the case of models within 2 AIC of each other, we chose the simplest model. Following this, elevation and valley were included as explanatory variables. The resulting model produced the AIC value 1,887.8 (Supplementary Table 1).

**Supplementary Table 1:** Shows the model selection process for the abundance model and corresponding AIC values.

| **Model** | **Elevation** | **Valley** | **Land cover** | **df** | **AIC** |
| --- | --- | --- | --- | --- | --- |
| 1 | X |  |  | 3 | 1938.47 |
| **2** | **X** | **X** |  | **4** | **1887.79** |
| 3 | X | X | X | 5 | 1888.48 |

The final model was defined as:

$Rat abundance=\beta_{0}+\beta_{1}elevation+\beta_{2}valley+S\left( x_{i} \right)+ Z_{i}$,

where $\beta_{0}$ is the intercept, and $\beta_{1}$ and $\beta_{2}$ are the covariates. The geostatistical model included the $S(x_{i})$ term, which was a stationary and isotropic Gaussian process with variance $\sigma^{2}$, accounting for structural spatial correlation, and $Z_{i}$, accounting for unstructured spatial variation, also normally distributed, with variance $\tau^{2}$. The $Z_{i}$ term can also account for small-scale spatial differences that are present at distances smaller than those measured and are therefore manifested as noise. The covariance of $\left( S\left( x \right), S\left( x^{'} \right) \right)= \sigma^{2}\rho\left( u_{i,j} \right)$, where $\rho\left( u_{i,j} \right)$ – the correlation between location $i, j$ when the exponential correlation function is taken – is $e^{-\frac{u}{\phi}}$. The exponential correlation function is when the value of $\kappa$ (the smoothness parameter) is 0.5, meaning that it has no derivatives, and $\phi$ is the scale parameter, which regulates how fast correlation goes to 0.

The unknown parameters that needed to be estimated were: $\alpha, \beta, \sigma^{2}, \tau^{2}$, and $\phi$, where 𝛼 and 𝛽 were the fixed-effects for the intercept and coefficients, and $\sigma^{2}, \tau^{2}$, and $\phi$ have been defined above. Because the likelihood function – needed to estimate the parameters – was intractable here, the Monte Carlo Markov chain (MCMC) simulation was used to estimate the likelihood of the parameters. The starting values for parameter estimation were provided as the coefficients from the generalised linear model (GLM) and were calculated statistically using a theoretical variogram for $\sigma^{2}, \tau^{2}$, and $\phi$. The MCMC was run with 10,000 simulations, with a burn-in of 2,000 and using every eighth simulation because the first simulations are not considered to be representative, and to discard correlated consecutive simulations. The practical range – which is the distance at which spatial correlation is less than 0.05, and therefore negligible – was estimated as approximately 3 times the exponential of $\phi$, by rearranging: $0.05=e^{-\frac{u}{\phi}}$. The confidence intervals for the parameters were calculated as:

$Parameter\pm1.96 SE$,

where $SE$ is the standard error.

# Details of shedding model

The components of the zero-inflation Gaussian model have been described below and consisted of 1) the probability of a rat being seropositive as a logistic regression ($Y_{1, i}$) and 2) the amount of shedding by seropositive rats using a linear mixed model ($Y_{2, i}$).

To carry out exploratory analysis, all the relevant variables were included into the full linear regression model for individual rat shedding, and stepwise elimination was done by removing variables and comparing the AIC sequentially to select the best model, prioritising the lowest AIC value, and simplicity, as with the abundance model (1). The resulting linear model had the AIC value of 339.4 (Supplementary Table 2). Although removing sex achieved the lowest AIC value (338.4 vs. 339.4), this covariate was not removed as sex is known to be an important explanatory factor determining individual rat characteristics, and the difference in AIC values was within 2 AIC (2).

**Supplementary Table 2:** Shows the model selection process for the shedding model and corresponding AIC values.

| **Model** | **Time**  **Month 1-12** | **Time**  **Month >12** | **Elevation**  **<40m** | **Elevation**  **>40m** | **Distance to sewer** | **Valley** | **Land cover** | **Age** | **Sex** | **Sexual activity** | **df** | **AIC** |
| --- | --- | --- | --- | --- | --- | --- | --- | --- | --- | --- | --- | --- |
| 1 | X | X | X | X |  |  |  | X |  | X | 8 | 338.40 |
| **2** | **X** | **X** | **X** | **X** |  |  |  | **X** | **X** | **X** | **9** | **339.38** |
| 3 | X | X | X | X |  |  | X | X | X | X | 10 | 339.87 |
| 4 | X | X | X | X |  | X | X | X | X | X | 12 | 340.35 |
| 5 | X | X | X | X | X | X | X | X | X | X | 13 | 342.20 |

For the zero-inflated Gaussian (3) portion of the model, exploratory analysis was done by including known explanatory variables of seropositivity in the model and backward elimination was carried out to decide the best model with the lowest AIC. The model producing the lowest AIC value included rat age and sexual activity (Supplementary Table 3; AIC value: 392.7). We chose to use age (AIC value: 395.2) alone because we favoured the simpler model, even though the resulting model had a slightly higher AIC value (Supplementary Table 3).

**Supplementary Table 3:** Shows the model selection process for the zero-inflated part of the shedding model and corresponding AIC values.

| **Model** | **Age** | **Sex** | **Sexual activity** | **Elevation**  **<40m** | **Elevation**  **>40m** | **df** | **AIC** |
| --- | --- | --- | --- | --- | --- | --- | --- |
| 1 | X |  | X |  |  | 4 | 392.70 |
| 2 | X | X | X |  |  | 5 | 394.70 |
| **3** | **X** |  |  |  |  | **3** | **395.19** |
| 4 | X |  | X | X | X | 6 | 393.35 |
| 5 | X | X | X | X | X | 7 | 395.34 |

$$Y_{i}=Y_{1, i}\times Y_{2, i}$$

$$P\left( Y_{1, i}=1 \right)= p_{i}$$

$$log\left( \frac{p_{i}}{1- p_{i}} \right)=\gamma_{0}+ \gamma_{1}rat age$$

$$Y_{2, i}\sim N(\mu_{i}, \sigma^{2})$$

$\mu_{i}= \beta_{0}+ \beta_{1}t+ \beta_{2}rat age+ \beta_{3}sex+ \beta_{4}sexual activity+\beta_{5}elevation+ \beta_{6}\max\left\{ elevation-40, 0 \right\}+ \beta_{7}\max\left\{ t-12, 0 \right\}+Z_{i}$,

where $p_{i}$ was the probability of a rat being seropositive, $\gamma_{0}$ and $\beta_{0}$were the intercept, $\gamma_{1}$ and $\beta_{1-7}$were the coefficients for the covariates included in the model, $Z_{i}$ was the error term, and $\mu_{i}$ and $\sigma^{2}$ were the mean and variance of log rat shedding. Time and elevation were included in the model with a spine to account for non-linear trends.

To check for residual spatial correlation after accounting for the covariate effects, an empirical variogram was plotted, which is defined as:

$$V\left( u_{i,j} \right)= {\frac{1}{2}(Z_{i}-Z_{j})}^{2},$$

where $Z$ are predicted independent residuals. In the presence of spatial correlation, the variogram should show an increasing trend, because the squared differences between the predicted residuals would be smaller at short distances than at larger ones. Because each pair of distance is often unique in such datasets, classes of distances were used here instead, and an average of these was plotted to allow greater confidence in the resulting variogram. The maximum distance to be estimated was obtained by taking half of the maximum distance between residuals. To ensure that any observed trend was not due to chance, envelopes were created, by permuting 1,000 variograms with no correlation. If the empirical variogram fell outside this envelope, this suggested the presence of residual spatial correlation. Supplementary Figure 2 shows the resulting variogram from this model and we concluded that there was no evidence residual spatial correlation, as it lies within the envelopes.


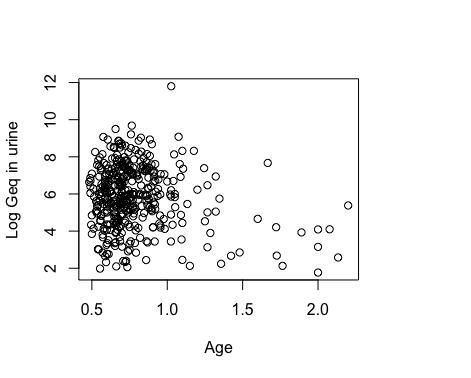
**Supplementary Figure 1:** Shows the variables associated with leptospire shedding plotted against the outcome (N=400). Figures A-E show the individual rat characteristics, and figures F-J show the environmental factors. The length-weight ratio of the rat has been used as a proxy for rat age and has been divided into bins for age groups as a categorical variable (A), and as a continuous variable (B). A spline was included to account for the non-linear trend seen in elevation at 40m (F), and at time 12 (J).

A

B


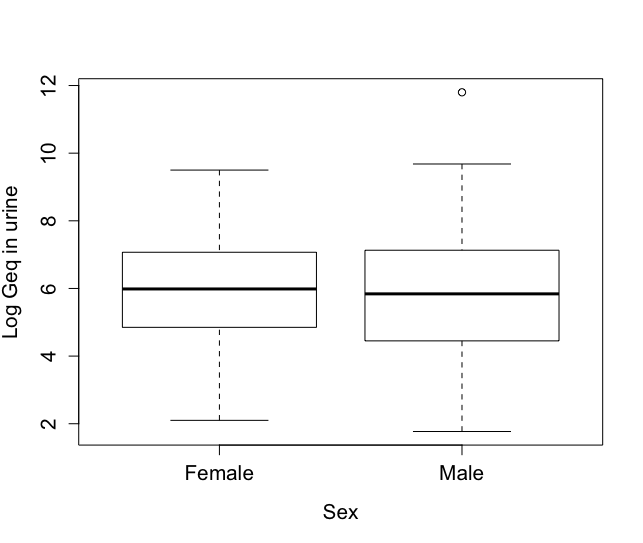

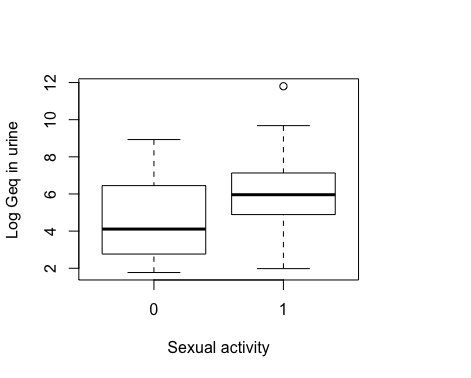

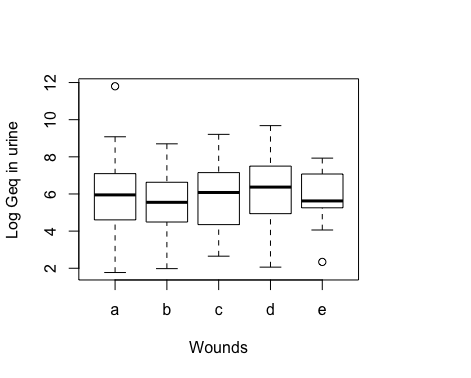


E


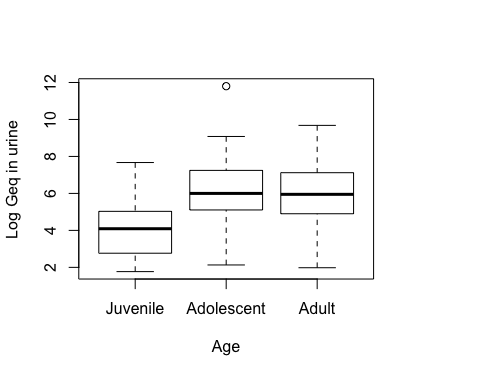


Young adult

D

C


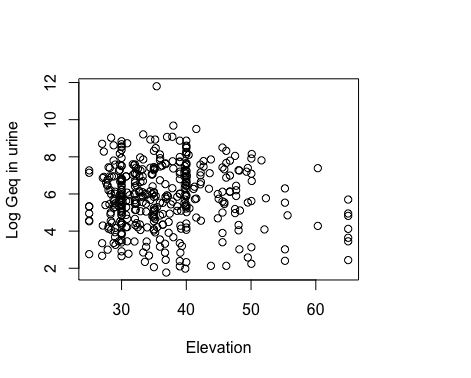

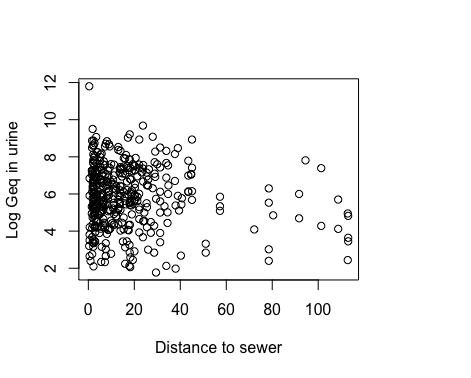

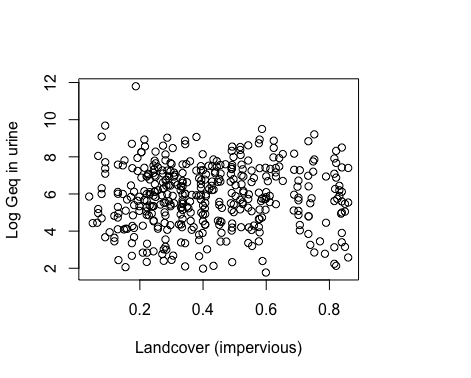


F

G


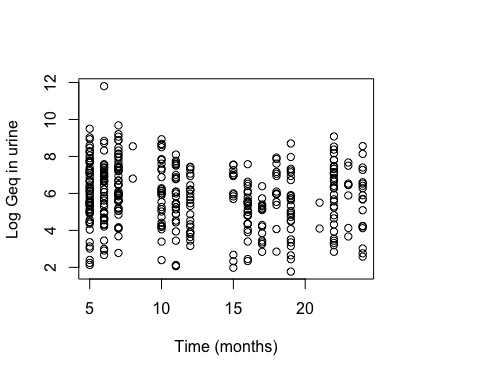

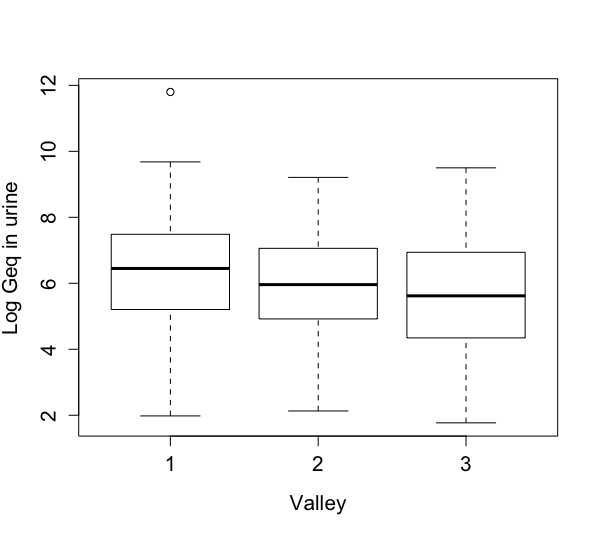


J

I

H

**Supplementary Figure 2:** Shows the variogram of the residuals from the final shedding model (solid line) and the envelopes representing 1,000 variograms with no correlation (dotted lines).

**
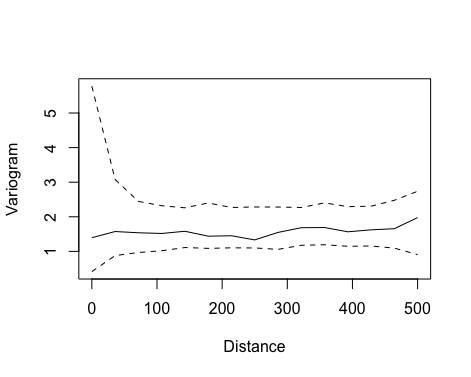
**

# Details of human model

To visualise explanatory variables with a binomial outcome, the empirical logit was calculated by grouping continuous variables into equally spaced bins, using the number of infected and uninfected individuals, and the formula for this was:

$log\frac{\mathrm{pos} + 0.5}{neg+0.5}$,

where, within each bin, $pos$was the number of seroconversions, and $neg$ was the number of seronegative individuals (4).

Univariate analysis between the chosen explanatory variables and the outcome was done using a generalised linear model (GLM). The relationship between infection event and leptospire shedding was explored by assigning estimates of ‘total shedding’ (individual rat shedding multiplied by rat abundance) to individuals based on their household coordinates. A mean of the prediction distribution was used to carry out exploratory analysis and model selection. Supplementary Figure 3 and 4 show the explanatory variables plotted against the empirical logit. The shedding variable was included linearly into the model, as well as rainfall, and landcover. Age and elevation were included using a spline at 32.5 and 22.5, respectively, to account for non-linear trends.

After including all the relevant variables into a GLM with a logit transformation, and performing backward-elimination, the final model, producing the lowest AIC value (Supplementary Table 4) of 1,218.0 was the following:

$Infection risk=\beta_{0}+ \beta_{1}shedding+ \beta_{2}age+ \beta_{3}sex+ \beta_{4}literacy+\beta_{5}rainfall+ \beta_{6}landcover+ \beta_{7}\max\left\{ age-32.5, 0 \right\}+ \beta_{8}floodwater$,

where, the log risk of infection was the outcome, $\beta_{0}$ was the intercept, and $\beta_{1}-\beta_{8}$ were the coefficients for the variables included in the model.

**Supplementary Table 4:** Shows the model selection process for the human infection model and corresponding AIC values.

| **Model** | **Elevation**  **<22.5m** | **Elevation**  **>22.5m** | **Rain** | **Land cover** | **Shedding** | **Sex** | **Age**  **<32.5** | **Age**  **>32.5** | **Literacy** | **Job** | **Sewer** | **Flood** | **df** | **AIC** |
| --- | --- | --- | --- | --- | --- | --- | --- | --- | --- | --- | --- | --- | --- | --- |
| **1** |  |  | **X** | **X** | **X** | **X** | **X** | **X** | **X** |  |  | **X** | **9** | **1217.99** |
| 2 | X |  | X | X | X | X | X | X | X |  |  | X | 10 | 1219.96 |
| 3 | X | X | X | X | X | X | X | X | X |  |  | X | 11 | 1221.80 |
| 4 | X | X | X | X | X | X | X | X | X |  | X | X | 12 | 1222.18 |
| 5 | X | X | X | X | X | X | X | X | X | X | X | X | 13 | 1223.47 |

To check for residual spatial correlation after accounting for the covariate effects, and if a geostatistical model was required, an empirical variogram was plotted (defined earlier). Supplementary Figure 5 shows the resulting variogram from this model and we concluded that there was no evidence residual spatial correlation, as it lies within the envelopes.

**Supplementary Figure 3:** Shows the relationship between the empirical logit and the continuous variables; age in years (A), elevation in metres (B), total rainfall experienced in mm (C), and the proportion of vegetative landcover within a 10m radius (D). These continuous variables have been split into equally bins and their midpoints have been plotted against the empirical logit. A spline was added at age 32.5, to account for the initially increasing, and then a plateauing trend after this point, which represented the midpoint of the 30-35 age category (few datapoints were available at the higher age groups; A). Elevation was also included in the final model using a spline, to account for the initially decreasing and then increasing trend at 22.5m, the midpoint of the 20-25m elevation category (B).


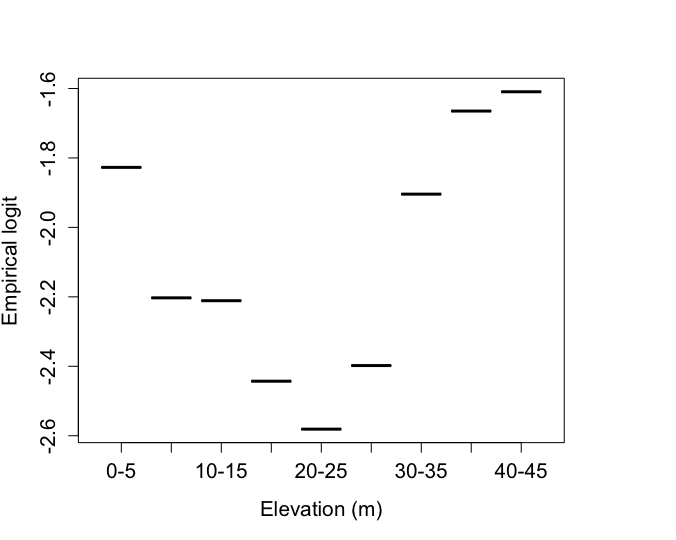
**
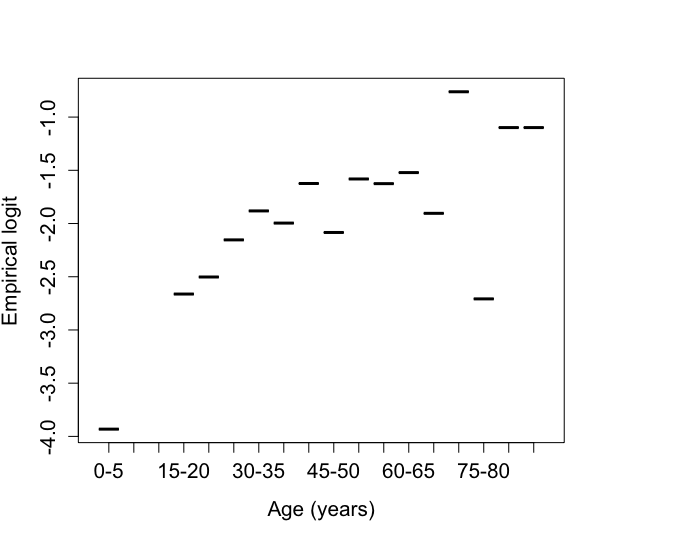
**

D

C

A

B

**
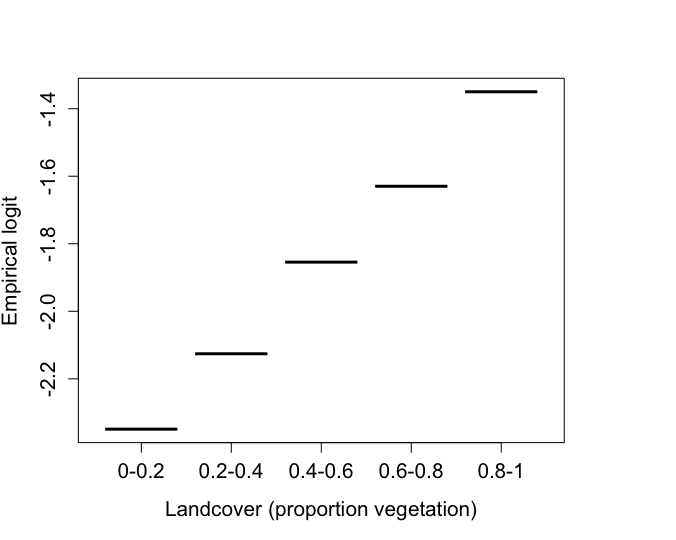

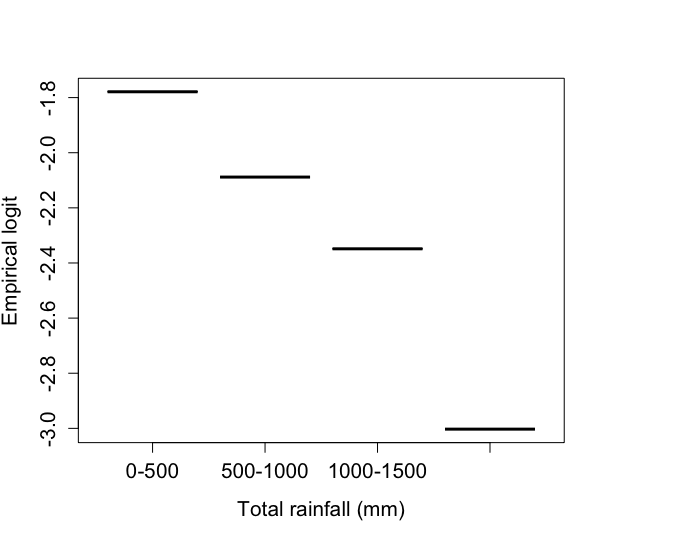
**

**Supplementary Figure 4:** Shows the relationship between leptospire shedding in space and the empirical logit. This was included linearly into the model.

**
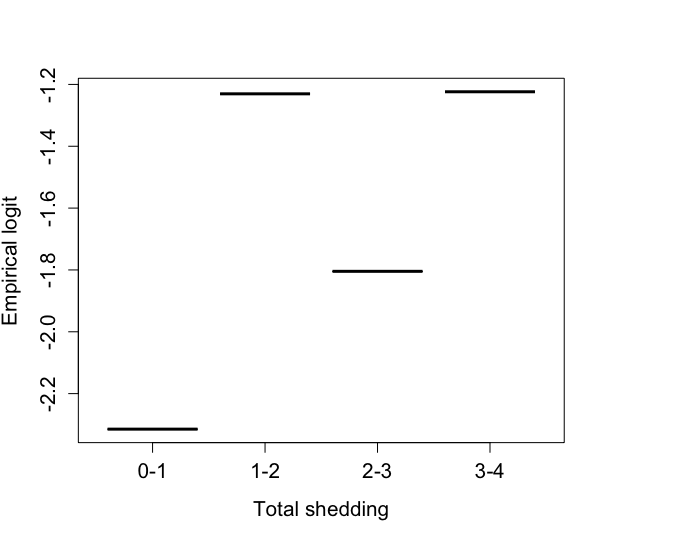
**

**Supplementary Figure 5:** Shows the variogram of the residuals from the final human infection model (solid line) and the envelopes representing 1,000 variograms with no correlation (dotted lines).

**
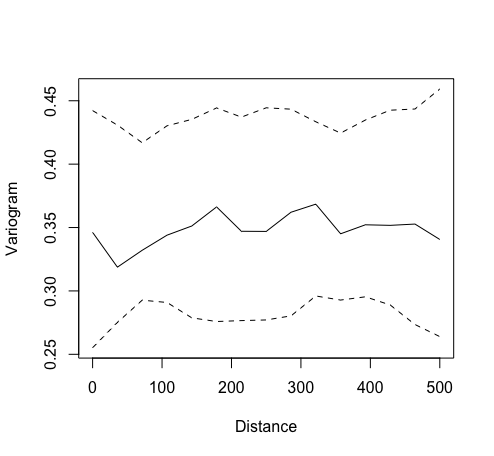
**

# Results

## Results of abundance model

Supplementary Table 5 shows the summary of the final abundance model, including the fixed and random effects.

**Supplementary Table 5:** Shows the summary of the binomial geostatistical analysis of predictors for rat abundance.

| **Fixed-effects** | **OR*** | **95% CI*** |
| --- | --- | --- |
| **Intercept** | **0.38** | **0.21, 0.66** |
| **Elevation** | **0.96** | **0.93, 0.99** |
| **Valley** |  |  |
| 1 | Ref | - |
| 2 | **0.36** | **0.20, 0.62** |
| 3 | **0.43** | **0.25, 0.74** |
| **Random-effects** | **Estimate** | **95% CI*** |
| Sigma^2^ | 2.04 | 1.66, 2.51 |
| Phi | 11.90 | 8.82, 16.07 |
| Tau^2^ | 0.18 | 0.11, 0.28 |

*Bold when p-value <0.05; OR, Odds ratio; CI, Confidence interval; Ref, Reference level.

Here, the estimate of the practical range was 35.7m, meaning that locations would be affected by what happened in this range, and correlation would be negligible at distances greater than the practical range, where locations could be considered independent. Additionally, the value of $\tau^{2}$ was smaller than that of $\sigma^{2}$, meaning there was more structured unexplained spatial correlation present compared to unstructured.

## Results from the individual shedding model

**Supplementary Table 6:** Shows the stratification of the total rat population included in the analysis by *Leptospira* colonisation and characteristics (N=461), and results from the univariate analysis, and mean shedding by the seropositive rat population (N=374).

|  | **PCR-positive rats**  **(N=374)^a^** | | | **PCR-negative rats (N=87)^a^** | |
| --- | --- | --- | --- | --- | --- |
| **Characteristic** | **N** | **Mean log_10_ Geq per ml of urine (95% CI)** | **Estimate**  **(95% CI)*** | | **N** |
| **Age** | | | | | |
| Juvenile | 22 | 4.05 (3.39, 4.71) | Ref | | 31 |
| Young adult | 81 | 6.06 (5.69, 6.42) | **2.00 (1.24, 2.77)** | | 24 |
| Adult | 271 | 5.90 (5.71, 6.09) | **1.85 (1.15, 2.55)** | | 32 |
| **Sex^a^** | | | | | |
| Female | 153 | 5.91 (5.66, 6.15) | Ref | | 38 |
| Male | 221 | 5.77 (5.54, 6.00) | -0.14 (-0.48, 0.21) | | 49 |
| **Wounds** | | | | | |
| Absent | 140 | 5.78 (5.49, 6.08) | Ref | | 60 |
| Very light | 72 | 5.58 (5.23, 5.92) | -0.21 (-0.68, 0.27) | | 12 |
| Light | 76 | 5.84 (5.45, 6.23) | 0.06 (-0.41, 0.53) | | 11 |
| Moderate | 58 | 6.20 (5.76, 6.64) | 0.42 (-0.09, 0.93) | | 4 |
| Serious | 28 | 5.90 (5.44, 6.36) | 0.12 (-0.56, 0.80) | | 0 |
| **Evidence of sexual activity** | | | | | |
| No | 25 | 4.41 (3.65, 5.18) | Ref | | 30 |
| Yes | 349 | 5.93 (5.76, 6.10) | **1.52 (0.85, 2.18)** | | 57 |
| **Valley** | | | | | |
| 1 | 85 | 6.24 (5.86, 6.63) | Ref | | 19 |
| 2 | 145 | 5.91 (5.66, 6.16) | -0.33 (-0.78, 0.11) | | 29 |
| 3 | 144 | 5.50 (5.22, 5.77) | **-0.74 (-1.19, -0.30)** | | 39 |
| **Landcover** | - | - | 0.06 (-0.76, 0.88) | | - |
| **Distance to sewer** | - | - | **-0.01 (-0.02, -0.00)** | | - |
| **Time** | | | | | |
| Month 1-12 | - | - | **-0.13 (-0.21, -0.05)** | | - |
| Month >12 | - | - | **0.16 (0.04, 0.28)** | | - |
| **Elevation** | | | | | |
| 0-40m | - | - | **0.04 (0.00, 0.09)** | | - |
| >40m | - | - | **-0.11 (-0.18, -0.04)** | | - |

^a^ 1 rat had undetermined sex and was not included here.

*Bold when p-value <0.05; CI, Confidence interval; Ref, Reference level; N, Number.

## Results of the model with individual abundance and shedding included separately

**Supplementary Table 7:** Shows the summary of the human infection multivariable logistic regression analysis of predictors with individual abundance and shedding included separately.

| **Characteristic** | **OR*** | **95% CI*** |
| --- | --- | --- |
| **Intercept** | **0.057** | **0.008, 0.415** |
| **Individual shedding** | 0.998 | 0.711, 1.401 |
| **Abundance** | 1.770 | 0.576, 5.440 |
| **Age** |  |  |
| 0-32.5 years old | **1.049** | **1.026, 1.072** |
| >32.5 years old | **0.958** | **0.927, 0.991** |
| **Sex** |  |  |
| Female | **Ref** | **-** |
| Male | **1.947** | **1.433, 2.645** |
| **Literacy** |  |  |
| Illiterate | Ref | - |
| Literate/under 10 | **0.657** | **0.445, 0.969** |
| **Rainfall experienced between paired samples (mm)** | **0.999** | **0.998, 1.000** |
| **Landcover** | **2.347** | **1.149, 4.797** |
| **Floodwater entered house** | **2.361** | **1.640, 3.398** |

*Bold when p-value <0.05; OR, Odds ratio; CI, Confidence interval; Ref, Reference level.

**References**

1. Akaike H. Factor analysis and AIC. *Psychometrika* (1987) 52:317–332. doi: 10.1007/BF02294359

2. Costa F, Wunder EA, De Oliveira D, Bisht V, Rodrigues G, Reis MG, Ko AI, Begon M, Childs JE. Patterns in Leptospira Shedding in Norway Rats (Rattus norvegicus) from Brazilian Slum Communities at High Risk of Disease Transmission. *PLoS Negl Trop Dis* (2015) 9:e0003819. doi: 10.1371/journal.pntd.0003819

3. Zhang X, Guo B, Yi N. Zero-Inflated gaussian mixed models for analyzing longitudinal microbiome data. *PLoS One* (2020) 15:e0242073. doi: 10.1371/journal.pone.0242073

4. Legler PR and J. *Beyond Multiple Linear Regression*. https://bookdown.org/roback/bookdown-BeyondMLR/ch-logreg.html [Accessed September 11, 2022]
